# Genetic diversity analysis and fingerprint construction of Korean pine (*Pinus koraiensis*) clonal seed orchard

**DOI:** 10.3389/fpls.2022.1079571

**Published:** 2023-01-16

**Authors:** Pingyu Yan, Zixiong Xie, Kele Feng, Xinyu Qiu, Lei Zhang, Hanguo Zhang

**Affiliations:** ^1^ State Key Laboratory of Tree Genetics and Breeding, Northeast Forestry University, Harbin, China; ^2^ Heilongjiang Academy of Forestry, Harbin, China

**Keywords:** Korean pine, SSR, genetic diversity, clone, fingerprint

## Abstract

Korean pine is a native tree species in Northeast China. In order to meet the needs of germplasm resource evaluation and molecular marker-assisted breeding of Korean pine, we collected Korean pine clones from 7 populations in Northeast China, analyzed the genetic diversity and genetic structure by SSR molecular marker technology and clustered them to revealed the inter- and intrapopulation differentiation characteristics of each clone. The fingerprint profiles of 161 Korean pine clones were also constructed. 77 alleles were detected for 11 markers, and 18 genotypes were identified on average for each marker. The PIC of the different markers ranged from 0.155-0.855, and the combination of PI and PIsibs for the 11 markers was 3.1 × 10^-8^ and 1.14 × 10^-3^, respectively. MANOVA showed that genetic variation existed mainly within populations, accounting for 98% of the total variation. The level of genetic differentiation among populations was low, with an average *Nm* between populations of 11.036. Genetic diversity is lower in the Lushuihe population and higher in the Tieli population. The 161 Korean pine clones were divided into 4 or 7 populations, and the 7 populations were not clearly distinguished from each other, with only the Lushuihe population showing partial differentiation. There is no significant correlation between the genetic distance of Korean pine populations and the geographical distance of their superior tree sources. This result can provide recommendations for future Korean pine breeding programs. The combination of 11 markers could completely distinguish 161 clones and establish the fingerprint. Genetic diversity of Korean pine clones from the 7 populations was abundant, and the genetic distances of individuals and populations were evenly dispersed. The fingerprint map can be used for the identification of Korean pine clones.

## 1 Introduction

Genetic diversity is the basis of evolution (Hughes et al., 2008) and provides the raw material for evolution of natural selection (Nevo, 1988; Zhang et al., 2022). Intraspecific genetic variation is the basis and most basic level of biodiversity (Pauls et al., 2013), and it is important for the evolution and conservation of species (Ellegren and Galtier, 2016). The level of genetic diversity within a population affects the productivity, growth and stability of that population (Hughes et al., 2008). Genetic diversity may not necessarily enable a population to persist, but reduced genetic diversity in a population may have long-term effects on its future evolution, as well as on its adaptive capacity in times of environmental change (Jump et al., 2009; Markert et al., 2010). The assessment of genetic diversity within and among populations is important for decision-making in genetic conservation programs, because studying the relationship between genetic diversity and fitness can predict the importance of genetic diversity for a given population (Eding et al., 2002). The genetic basis of a breeding population determines the genetic quality and long-term potential of breeding programs and products (Ivetić et al., 2016). The size of parental populations determines the level of genetic diversity in new stands (Flint-Garcia, 2013), so it is in our best interest to maintain diversity and promote systematic redundancy and resilience (Ledig, 1992). To avoid population genetic bottlenecks and maintain maximum effective population size, appropriate sampling strategies can maximize increase genetic diversity in the population of seed production (Ivetic et al., 2016). Regular monitoring of trends in genetic diversity utilization in breeding programs can provide breeders with options for developing new varieties and hybrids (Govindaraj et al., 2015; Jin et al., 2016).

Korean pine (*Pinus koraiensis*), a genus of pine in the family Pinaceae, National Key Conserved Wild Plants of Grade II in China, is a native species in northeast China (Lim, 2012). Traditionally, Korean pine is a good tree species capable of providing wood, pulp and oil. In addition, the seed of Korean pine is the most popular pine nut due to its nutritional value (Yoon et al., 1989; Wolff et al., 2000), high amounts of crude protein, crude fat, polysaccharides and crude fiber as well as vitamins, minerals and trace elements (Ca, P, Mn, Co, Cu and Zn) (Nergiz and Donmez, 2004; Zadernowski et al., 2009). The market demand for superior Korean pine seeds has promoted the development of Korean pine clones seed orchard, which were established in China as early as the early 1960s, and the technical system for the creation from fringe picking to seedlings management was proposed in the 1970s. Subsequently, Korean pine clones seed orchards were established in many places in northeast China to improve the genetic quality of Korean pine seeds that can be used for afforestation. At the same time, research on productivity techniques, flowering and fruiting patterns in Korean pine seed orchards is also being conducted (An et al., 1992). These excellent Korean pine resources have become important conventional breeding materials and are used in traditional breeding studies, including analysis of fruiting traits, selection of superior clones, analysis of seed traits, nutrient composition, variation studies of seed traits, genetic diversity analysis and studies on phenotypic diversity of needles and cones in Korean pine seed orchard (Zhang et al., 2015b; Tong et al., 2019; Weihuai et al., 2019; Pingyu et al., 2020; Qianping, 2020; Longhai et al., 2021). In addition, studies on the reaction conditions of ISSR, SSR and SRAP in Korean pine, laying the foundation for genetic differentiation of Korean pine populations based on molecular markers (Feng et al., 2004; Feng et al., 2010; Zhao et al., 2010).

Follow-up surveys conducted to confirm clones have generally shown that mislabeling of seed orchard divisions is relatively common (Wheeler and Jech, 1992). Plant varieties are often identified by morphology, traditionally. However, it is difficult to identify different clones morphologically, because there is little morphological variation among clones, and some morphological appearances are susceptible to environmental factors. The limitations of genetic markers for phenotype have led to the development of more effective-directly DNA-based markers called molecular markers, which is specific DNA fragments representing genome-level differences (Agarwal et al., 2008). Microsatellite is ideal for identifying individuals and studying genetic diversity, due to their ubiquity, reproducibility, a high level of polymorphism, co-dominant and high levels of transferability (Guan et al., 2019; Lv et al., 2020; Nn et al., 2020; Carletti et al., 2021). Therefore, SSR has been used for genetic diversity studies, genetic linkage, and fingerprinting of many important economic tree species, such as Date palm (*Ziziphus jujuba* Mill.), Poplar (*Populus* L.), and Pear (*Pyrus* spp) (Liang et al., 2005; Gao et al., 2012; Ma et al., 2012), as well as pines such as Masson pine (*Pinus massoniana*) (Afeng, 2005) and Camphor pine (*Pinus sylvestris* var. *mongolica*) (Huili et al., 2022).

In this study, we collected 161 clones from 7 Korean pine seed orchards in northeastern China. 11 SSR genotyping data of 161 clones of Korean pine were obtained by capillary electrophoresis. The fingerprint map of Korean pine clones was established, which provides a strong guarantee of technology for resource sharing and the distribution application of superior clones, and has important value in property protection and promotion of superior seed. In addition, the genetic diversity and genetic structure of Korean pine clones seed orchard are evaluated and systematically described, which can help improve the utilization efficiency of Korean pine resources, guide the development of further breeding strategies, and provide a basis for the scientific utilization of Korean pine germplasm resources.

## 2 Materials and methods

### 2.1 Plant materials and DNA extraction

In this study, a total of 161 clones were collected from 7 Korean pine seed orchards in Heilongjiang and Jilin Province, whose superior tree (refers to individuals with excellent growth, timber and resistance adaptations in natural or planted forests with similar environmental conditions, such as the same stand conditions, the same forest age and the same forestry measures) originated from 6 sites in Changbai Mountains and Xiaoxinganling, the main distribution areas of Korean pine (**Table 1**). Total of 805 samples collected, with 5 ramets has collected from each clone. Annual conifers of Korean pine were collected and snap-frozen in liquid nitrogen for DNA extraction.

**Table 1:** Summary of material source information.

Population	Source of Superior Tree	Location (°)	Elevation (m)	Number of clones	Clones
Bohai	Xiaobeihu	N 44.21; E 128.56	743	18	BH1, BH6, BH8, BH16, BH26, BH38, BH45, BH51, BH61, BH63, BH66, BH67, BH69, BH70, BH71, BH73, BH92, BH93
Hegang	Wuying	N 48.24; E 129.25	547	26	HG3, HG4, HG7, HG8, HG9, HG10, HG11, HG12, HG14, HG15, HG17, HG21, HG24, HG25, HG26, HG27, HG28, HG29, HG30, HG31, HG39, HG40, HG44, HG46, HG47, HG51
Lushuihe	Lushuihe	N 42.47; E 127.78	775	21	LSH21, LSH22, LSH25, LSH38, LSH96, LSH99, LSH105, LSH106, LSH117, LSH127, LSH132, LSH139, LSH161, LSH162, LSH165, LSH169, LSH179, LSH193, LSH194, LSH331, LSH428
Weihe	Hebei	N 48.08; E 130.31	458	25	WH025, WH091, WH112, WH114, WH115, WH116, WH117, WH136, WH137, WH138, WH139, WH140, WH141, WH142, WH145, WH146, WH147, WH148, WH187, WH188, WH192, WH194, WH196, WH198, WH200
Linkou	Wuying	N 48.24; E 129.25	547	25	LK6, LK10, LK11, LK12, LK13, LK14, LK15, LK16, LK17, LK18, LK19, LK24, LK25, LK26, LK27, LK79-1, LK79-4, LK79-5, LK79-9, LK79-11, LK79-13, LK79-33, LK79-35, LK79-36, LK79-37
Tieli	Langxiang	N 46.95; E 128.87	332	22	TL1006, TL1018, TL1024, TL1054, TL1068, TL1080, TL1090, TL1091, TL1102, TL1105, TL1112, TL1140, TL1149, TL1185, TL1194, TL1198, TL1204, TL1212, TL1270, TL1271, TL1298, TL1357
Sanchazi	Sanchazi	N 42.63; E 126.85	601	24	SCZ113, SCZ114, SCZ115, SCZ116, SCZ117, SCZ119, SCZ120, SCZ121, SCZ122, SCZ123, SCZ124, SCZ125, SCZ126, SCZ127, SCZ129, SCZ130, SCZ131, SCZ132, SCZ133, SCZ134, SCZ135, SCZ136, SCZ137, SCZ138

Total DNA of Korean pine samples was extracted using the DP-320 Plant Genome Extraction Kit (Tiangen, Beijing, China). The integrity of genomic DNA was examined using a 1% agarose gel, and DNA concentration and quality were examined using Micro-Spectrophotometer (Bio-DL, Shanghai, China.) after extraction. The concentration of each DNA sample was diluted to 10 ng/μL and stored at -20°C.

### 2.2 SSR primer selection and genotyping

A total 142 primer pairs from the published SSR primers of 7 species of Pinaceae (*Pinus taeda*, *Pinus albicaulis*, *Pinus dabeshanensis*, *Pinus armandii*, *Pinus koraiensis* and *Pinus massoniana*) were selected and synthesized by Sangon Biotech (Shanghai) Co., Ltd., Shanghai, China (Liewlaksaneeyanawin et al., 2004; Echt et al., 2011; Yu et al., 2012; Dou et al., 2013; Xiang et al., 2015; Zhouxian et al., 2015; Zhang et al., 2015a; Dong et al., 2016; Lea et al., 2018; Li et al., 2020). Ten samples of DNA were randomly selected for polymorphism screening of synthesized primers. A PCR system was performed on DNA Engine thermal cycler (Biometra, Ilmenau OT Langewiesen, Germany) in 20μl volumes containing 0.5 μM each of forward and reverse primers, 200 μM dNTP, 2.0 μL 10×buffer, 2 U Taq DNA polymerase (TransGen Biotech Co., Beijing, China), and around 10 ng DNA. The PCR program was as follows: 3 min at 94°C, 35 cycles of 30 s at 94°C, 30 s at *Tm* (**Table 2**), and 15 s at 72°; and a final extension at 72°C for 7 min.

**Table 2:** SSR primer information of Korean pine.

Locus	Primer Sequence	Motif	Tm (°C)	Size (bp)	Fluorescent dye	Reference
p49	F:GAGATGAGCGAATCTGGG	(AAG)7	52	261	FAM	Zhang et al., 2015b
	R:TACAAGTTCCACCTACGG					
p70	F:CAACATCGCCAATGACTC	(CTCA)6	54	294	FAM	
	R:CCTACCTACGCTCTGCTC					
p72	F:TGGGTTACCACCTTTAGC	(GCT)6	52	193	HEX	
	R:CAATCAGAGTCTGGAGCA					
p79	F:CCACCGCCAAGTCCATTA	(CAA)7	55	190	HEX	
	R:GCTTTGTTAGCCGTCCAG					
p82	F:GGAAGATGAATCGCAAACC	(GCG)6	54	280	ROX	
	R:ACACCCGCCTGAAGAGCA					
EPD11	F:GTGGATGCAATGAAGAAAAACAT	(AGG)6	60	139	TAM	Xiang et al., 2015
	R:ACGAATTGCAAAACTGCATAACT					
NFPK-34	F:AACCCACAGAAAGCTGAGGA	(TAA)6	60	221	TAM	Li et al., 2020
	R:CACCCCTGAACAGAGAGGAG					
P6*	F:TCAAATTACCAGACAATAA	(TA)3(GT)15	55	125	FAM	Yu, 2012
	R:GAATTCGCCAATGAAATCA					
P45*	F:CTTACATTTTGCTGCTTTTC	(TG)16(AG)17	55	173	HEX	
	R:TTGTCAGTTTTAGGTTGGAT					
P51*	F:CCTAAGAGCAATGTAAAATG	(AG)15	55	204	TAM	
	R:AGCTTGACAACGACTAACT					
P52*	F:CCATCCTTCAAATTTTCCT	(AG)26	56	138	ROX	
	R:GCCATTCTTTCTACCACTT					

The PCR products were then detected by 7% PAGE, and 11 SSR markers with good reproducibility and significant polymorphism were selected finally. Forward primer of each marker was labelled at the 5’ end with fluorescent dye HEX, 6-FAM, ROX, or TAM. PCR was performed under light-protected conditions with the same reaction system as above. All PCR products were sent to Sangon Biotech (Shanghai) Co., Ltd., Shanghai, China for capillary electrophoresis genotyping by ABI 3730XL (Applied Biosystems, Foster City, CA, USA) and the identification genotype data were collected for subsequent analysis.

### 2.3 Data analysis

#### 2.3.1 Analysis of marker polymorphism and identification power

The DNA polymorphism information was processed into a data matrix, and the data matrix was converted into various formats by DataFormater 2.7 for further analysis (Wenqiang et al., 2016). Genetic parameters such as number of alleles (*Na*), number of effective alleles (*Ne*), Shannon diversity index (*I*), observed heterozygosity (*Ho*) and expected heterozygosity (*He*) of each primer was calculated using Popgen 32 (Li et al., 2003), primer polymorphism information content (*PIC*) was calculated using PowerMarker V3.25 (Liu and Muse, 2005), and primer identity probabilities (*PI*) and random identity probabilities (*PIsibs*) were calculated using GenAIEX 6.51b2 (Peakall and Smouse, 2006). Significant deviations from both the Hardy-Weinberg equilibrium (*HWE*) and linkage disequilibrium (*LD*) between all pairs of SSR loci were identified by Genepop v4.2 (Raymond and Rousset, 1995).

#### 2.3.2 Genetic structure and genetic diversity analysis

GenAIEX 6.51b2 was used to calculate the number of alleles (*Na*), Shannon diversity index (*I*), number of effective alleles (*Ne*), number of more than 5% alleles (*Na, F>5*), observed heterozygosity (*Ho*), expected heterozygosity (*He*), unbiased expected heterozygosity (*uHe*), F-fixed index (*F*), and number of private loci (*NPA*) for each population (Peakall and Smouse, 2006). MANOVA, principal coordinates analysis (PCoA) and generation of interpopulation genetic differentiation coefficient (*Fst*) and gene flow (*Nm*) matrices were performed using GenAIEX 6.51b2 to delineate genetic variation between and within populations (Peakall and Smouse, 2006). Genetic distance matrix of clones clustering maps was generated by NtSys 2.10e and used for constructing a neighbor-joining dendrogram in MEGA 11 (Tohme et al., 1996; Tamura et al., 2011). Neighbor-joining dendrogram between populations was also constructed in MEGA 11 based on Nei genetic distance (Tamura et al., 2011).

Based on the latitude and longitude of the source location of superior tree, the geographical distance between the source locations of superior tree was calculated by the following formula:


$$\text{d}=\text{R}{*}^{\text{arcos}}\left[\text{cos}\left(\text{Y}1\right){*}^{\text{cos}}\left(\text{Y}2\right){*}^{\text{cos}}\left(\text{X}1-\text{X}2\right)+\text{sin}\left(\text{Y}1\right){*}^{\text{sin}}\left(\text{Y}2\right)\right]$$

R is the radii of the earth (6371.0 km);

X1, X2, Y1, Y2 are two location coordinates radians;

Radians= coordinates * Π/180;

SPSS v19.0 software was used to detect the correlation between geographic distance and genetic distance among the 6 superior tree sources.

The genetic structure was investigated in software STRUCTURE v2.3.4 using an admixed model with 100,000 burn-ins followed by 100,000 iterations (Ravelombola et al., 2018). Markov Chain Monte Carlo iterations run 10 times of a number (K = 2-18) of genetically homogeneous clusters. The operation results were imported into the Structure Harvester website (https://taylor0.biology.ucla.edu/structureHarvester/) (Earl and vonHoldt, 2012), and the optimal K values were selected according to the method of Evanno et al. (Evanno et al., 2005).

#### 2.3.3 Fingerprint mapping construction

The fingerprint map of 161 clones was generated by combining the 11 pairs of SSR primers obtained from screening, sorting them in order from smallest to largest according to the size of the target fragment. The clone genotypes were coded using letters and arranged in a certain order to obtain the clone gene code. The name, scientific name and location of the clone, the source of the superior tree and the fingerprint code were organized into a separate Excel and uploaded to the online platform (https://cli.im/) to obtain the corresponding QR code for each clone (Li et al., 2022).

## 3 Result

### 3.1 Analysis of SSR marker polymorphism and discriminatory ability

There were 55 combinations of loci in the whole population, of which 4 pairs (7.27%) had LD between loci combinations at the significance level of P<0.001. The results of primer genetic analysis (**Table 3**) showed that a total of 75 alleles were detected at the 11 SSR loci, of which 33.004 were effective alleles, with the mean value of major allele frequency was 0.622. 186 genotypes were identified, with an average of 16.909 genotypes per marker, and the observed heterozygosity and expected heterozygosity on average were 0.451 and 0.514, respectively. The mean values of Shannon diversity index and Nei diversity index were 1.094 and 0.512, respectively, indicating the high genetic diversity among clones. The polymorphism information content (*PIC*) of 11 markers ranged from 0.155 to 0.855, among which 10 markers showed moderate or high polymorphism relatively. All markers can effectively analyze the genetic structure and genetic diversity of Korean pine clones.

**Table 3:** Genetic diversity parameters of 11 SSR marker.

Locus	MAF	Na	Ne	N	Ho	He	Shannon	Nei	PIC	HWE	PI	PIsibs
p49	0.665	4	1.831	6	0.503	0.455	0.698	0.454	0.362	NS	0.418	0.646
p70	0.693	4	1.901	7	0.391	0.476	0.845	0.474	0.428	NS	0.319	0.590
p72	0.845	3	1.374	4	0.273	0.273	0.524	0.272	0.251	NS	0.545	0.748
p79	0.736	5	1.680	6	0.404	0.406	0.705	0.405	0.347	***	0.331	0.599
p82	0.832	4	1.400	5	0.186	0.287	0.530	0.286	0.256	***	0.533	0.737
EPD11	0.627	4	2.036	8	0.528	0.510	0.860	0.509	0.431	NS	0.321	0.580
NFPK-34	0.907	2	1.203	3	0.124	0.170	0.310	0.169	0.155	NS	0.593	0.773
P6*	0.640	9	2.297	22	0.534	0.566	1.287	0.565	0.543	NS	0.203	0.513
P45*	0.245	16	7.362	48	0.708	0.867	2.256	0.864	0.851	***	0.028	0.320
P51*	0.252	13	7.562	48	0.745	0.871	2.239	0.868	0.855	***	0.029	0.322
P52*	0.401	11	4.358	29	0.559	0.773	1.781	0.771	0.745	***	0.076	0.383
Mean	0.622	6.818	3.000	16.909	0.451	0.514	1.094	0.512	0.475		0.309	0.565
Total	–	75	33.004	186	–	–	–	–	–		3.1×10^-8^	1.14×10^-3^

^***^Denotes Significant departure from Hardy-Weinberg equilibrium at P<0.001. NS denotes meet Hardy-Weinberg equilibtium.

**Figure 1:**
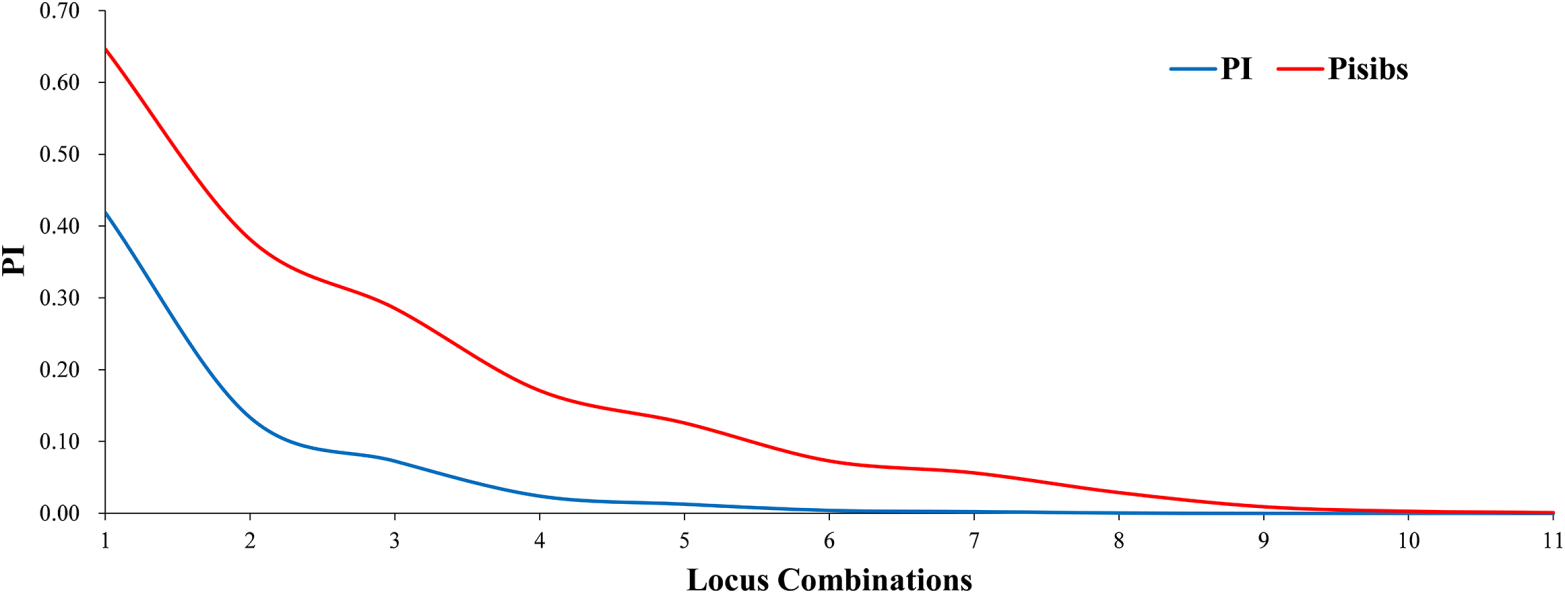
Identification ability of SSR markers in Korean pine clones.

Two key statistical values, PI and PIsibs, were calculated to assess the ability to identify 11 markers for Korean pine clones (**Table 3**). PI for each molecular marker ranged from 0.028-0.593 with a mean value of 0.393. PIsibs is often defined as the upper PI limit, and the PIsibs of the 11 SSR markers ranged from 0.320-0.773 with a mean value of 0.565. The cumulative probability of identity of markers according to the obtained data (**Figure 1**), PI tended to 0 when the number of marker combinations is 7 and PIsibs tended to 0 when the number of marker combinations is 11. Assuming that all marker loci are independent of each other, the probability of two random Korean pine clones having the exact same multi-locus genotype combination among all 11 molecular markers is estimated to be 3.1×10^-8^, and the combined PIsibs was 1.14×10^-3^. 161 Korean pine clones could be considered to be completely distinguished by the 11 SSR markers. The above results prove that the combination of these markers not only had high polymorphism but also showed a strong potential for fingerprint recognition.

### 3.2 Analysis of genetic structure and genetic diversity

#### 3.2.1 Analysis of genetic variation among populations

MANOVA was performed to determine variate characteristics of the 7 populations, and the results showed that (**Table 4**): population genetic differentiation coefficient (*Fst*) was 0.044 (P< 0.001), indicating a low level of genetic differentiation among populations. Genetic variation existed mainly within populations, accounting for 98% of the total variation, and the incidence of genetic variation among populations was only 2%. All of which indicated that there were extensive exchanges of genetic resources within each population. The level of genetic differentiation between populations was low, while the genetic variation within populations was much higher than that between populations. The inbreeding coefficient (*Fis*) was 0.078 (*Fis* > 0), indicating the presence of homozygous excess and the presence of interpopulation inbreeding.

**Table 4:** MANOVA for the population of Korean pine clones.

Source	DF	SS	MS	Variance component	Variance component/%	Fit	Fis	Fst
Among Pops	6	1244.936	207.489	2.767	2			
Within Pops	154	22172.480	143.977	143.977	98			
Total	160	23417.416		146.745	100	0.117 ^***^	0.078 ^***^	0.044 ^***^

^***^Denotes significant differences at P<0.001.

**Figure 2:**
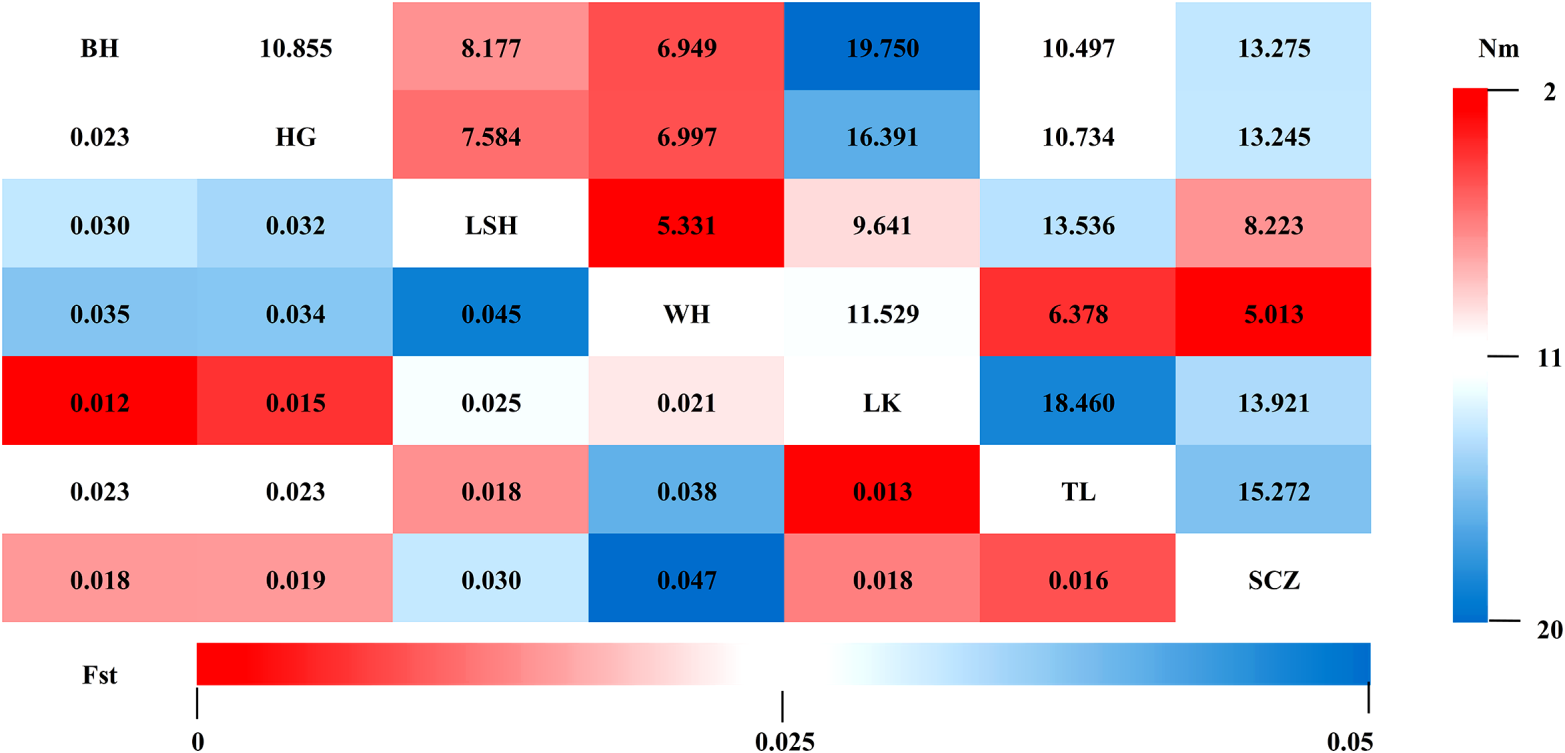
Genetic differentiation coefficients (lower left) and gene flow (upper right) between populations. (BH, Bohai; HG, Hegang; LSH, Lushuihe; WH, Weihe; LK, Linkou; TL, Tieli; SCZ, Sanchazi).

**Figure 3:**
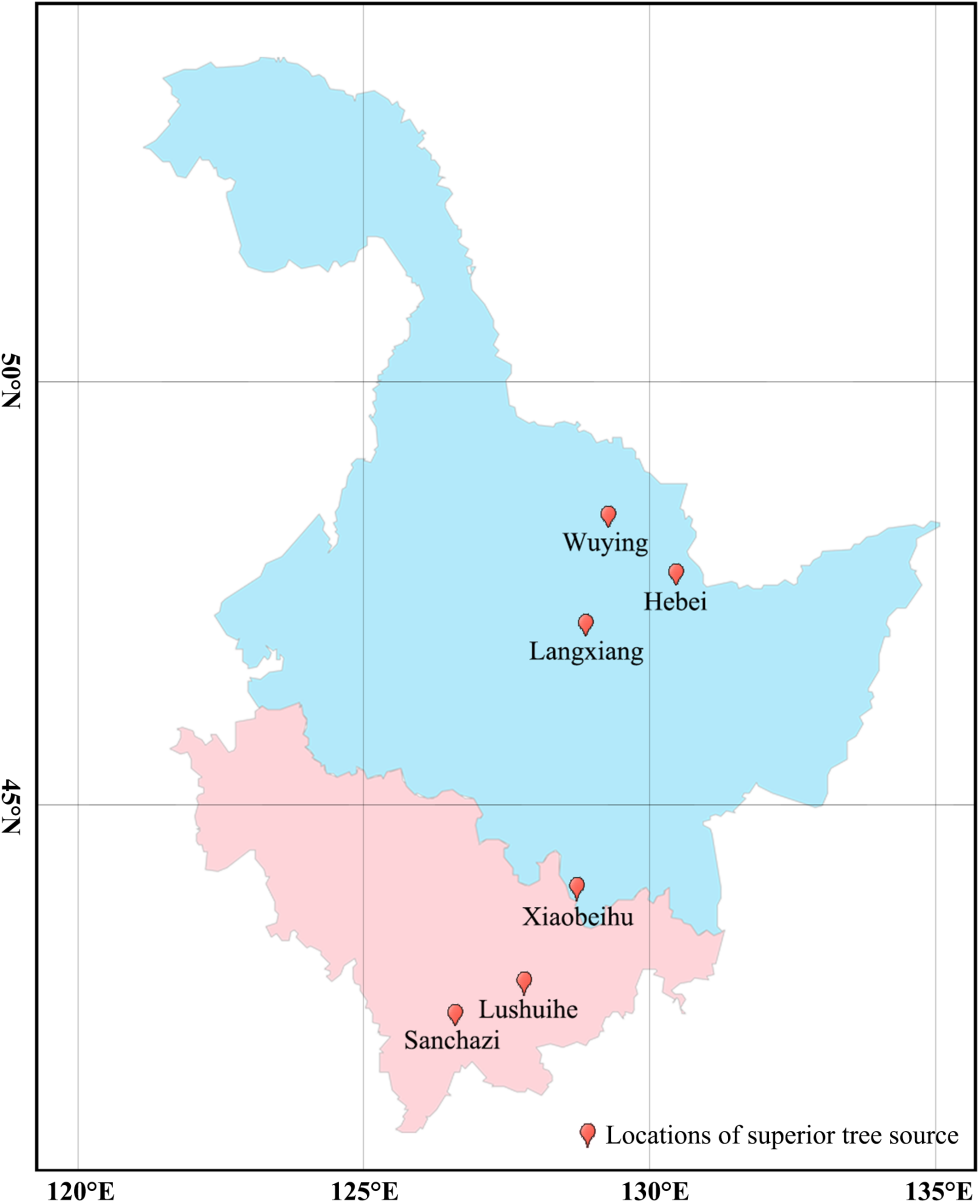
Geographical distribution of superior tree source of Korean pine populations. (Light blue indicates Heilongjiang Province and light purple indicates Jilin Province. The geographic conditions for each site can be found in **Table 1**).

*Fst* and *Nm* between two populations were calculated for seven populations to reveal genetic differences and gene flow among different populations of Korean pine clones. The results showed that (**Figure 2**): the *Fst* ranged from 0.012-0.047 with an average of 0.025, and the *Nm* ranged from 5.013-19.750 with an average of 11.036 among different populations, indicating that the genetic differentiation range among populations was small and there was a high frequency of genetic exchange. The highest *Fst* and the lowest *Nm* were found between the Lushuihe and Weihe, which may be due to the long geographical distance between Lushuihe and Hebei, the superior tree source of these two populations (**Figure 3**).

#### 3.2.2 Analysis of genetic diversity within populations

To assess genetic diversity and genetic differentiation of these 7 populations, genetic diversity analysis was performed and results showed that (**Table 5**): the level of genetic differentiation within 7 populations did not vary significantly, with Tieli population having the highest genetic diversity and the highest number of alleles, Shannon diversity index, observed heterozygosity at 55, 1.087, 0.479 respectively. the lowest Shannon diversity index and observed heterozygosity was in Lushuihe population with 0.915, 0.473 respectively.

**Table 5:** Genetic parameters of 7 Korean pine populations.

pop	Na	Ne	Na(F≥5%)	NPA	Shannon	Ho	He	uHe	F
Bohai	52	26.996	38.000	1	0.941	0.444	0.460	0.473	0.035
Hegang	52	30.149	38.000	0	1.002	0.448	0.505	0.515	0.083
Lushuihe	52	29.745	34.000	2	1.009	0.455	0.518	0.531	0.106
Weihe	53	31.431	36.000	0	0.915	0.473	0.437	0.445	-0.115
Linkou	52	31.084	39.000	1	1.001	0.425	0.488	0.498	0.119
Tieli	55	33.212	39.000	3	1.087	0.479	0.536	0.548	0.128
Sanchazi	53	30.790	38.000	2	1.012	0.436	0.502	0.512	0.074
Mean	52.714	30.487	37.429	1.286	0.995	0.451	0.492	0.503	0.061

The fixation index (*F*) ranged from -0.115 (Weihe) to 0.128 (Tieli), with an average of 0.061. F>0 indicates heterozygote deficiency, over-purity and inbreeding in Korean pine populations. Overall, the Tieli population showed high genetic diversity, while the Weihe population showed relatively lower genetic diversity, and no inbreeding was detected in this population.

**Figure 4:**
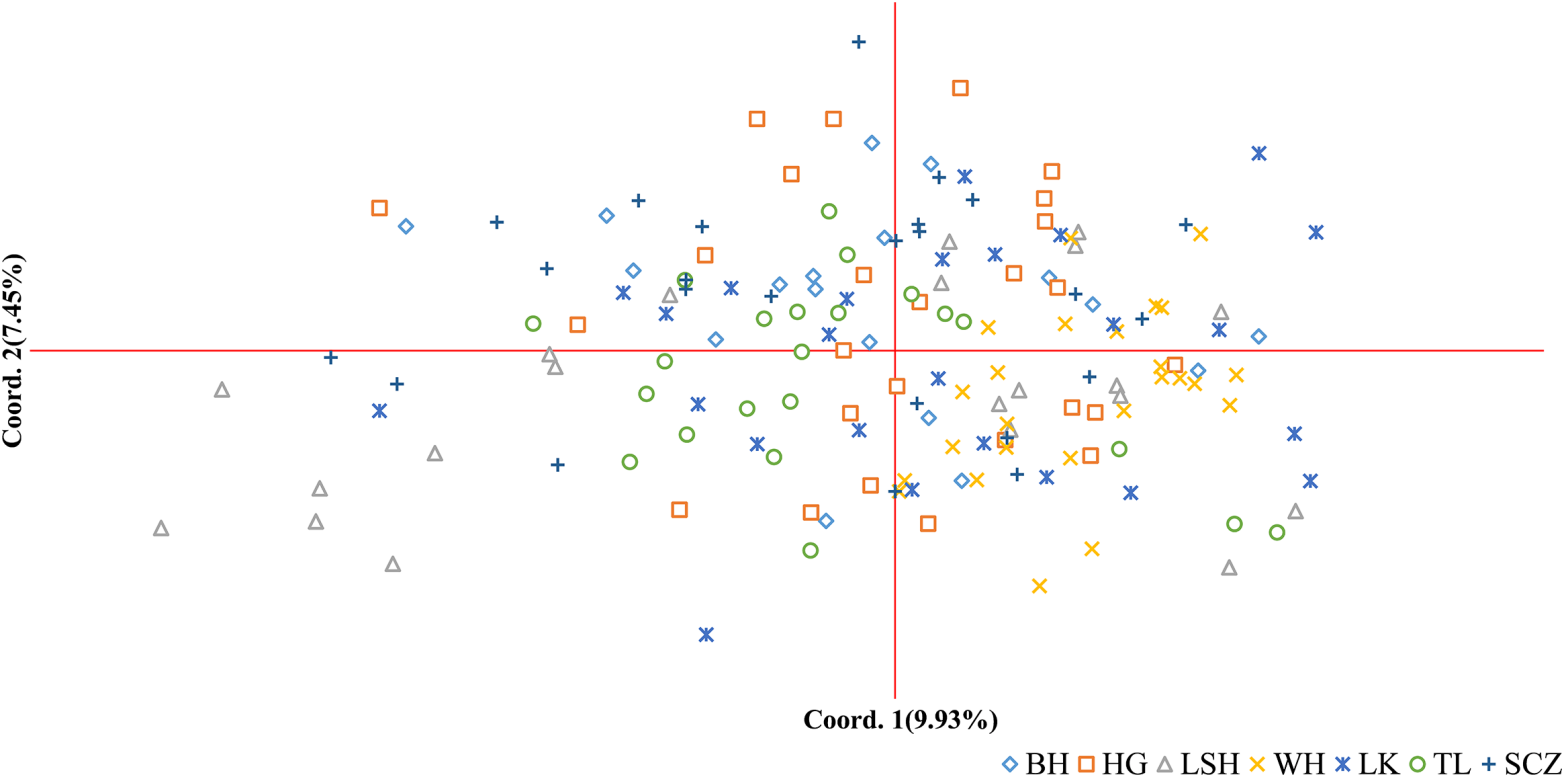
Principal coordinates analysis (PCoA) of Korean pine clones. (BH, Bohai; HG, Hegang; LSH, Lushuihe; WH, Weihe; LK, Linkou; TL, Tieli; SCZ, Sanchazi).

The results of principal coordinate analysis (PCoA) of Korean pine clones from 7 populations showed that Coordinates 1 explained 9.93% of the variation and Coordinates 2 explained 7.45% of the variation, indicating that each of the above molecular markers has a high degree of independence. There is a high degree of distribution overlap among populations in the figure, with the Lushuihe population having an extensive distribution and some clones showing relative independence, while the other populations are relatively clustered, with the Linkou population being more dispersed. There is some genetic divergence between the Weihe population and the Tieli population, and the distribution range of Weihe is the smallest, indicating that the genetic diversity of clones within the Weihe population is low (**Figure 4**), which is similar to the results of the Shannon diversity index in **Table 5**.

#### 3.2.3 Analysis of cluster and genetic structure

**Figure 5:**
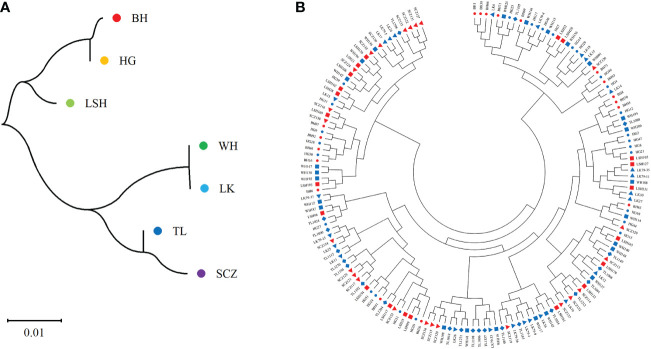
Neighbor-joining tree of populations and clones. **(A)** Neighbor-joining tree of 7 populations. **(B)** Neighbor-joining tree of 161Korean pine clons. (BH, Bohai; HG, Hegang; LSH, Lushuihe; WH, Weihe; LK, Linkou; TL, Tieli; SCZ, Sanchazi).

The results of cluster analysis among populations by the Nei genetic distance matrix showed (**Figure 5A**): The genetic distance among populations was small and the level of genetic differentiation was low, which was consistent with the results of MANOVA. The genetic distance between Bohai and Hegang, Lushuihe and Linkou was similar respectively, but the genetic distance of Weihe was far from Sanchazi. Lushuihe and Sanchazi were more independent, which was similar to the results of PCoA. However, it is worth noting that Hegang and Linkou have the same source location of superior tree, but they do not have the closest genetic relationship with each other. The correlation analysis between Nei genetic distance and geographic distance revealed that the Person coefficient was 0.075 (P=0.704), indicating the insignificant correlation between genetic distance and geographic distance of their superior tree sources.

The genetic distances of 161 clones were calculated by NTsys 2.10e software, and the results of clustering using MEGA showed that (**Figure 5B**): the clones from different sources were not clearly separated from each other, and the clones in each cluster did not come from the same location or the same superior source. The clones from different places were dispersed in each cluster. Clustering results did not correlate significantly with the location of the clones. The above results indicate that there is a high degree of gene exchange among populations and little genetic differentiation among populations. However, clones from Changbai Mountain are highly distributed on the left side of the clustering map, while clones from Xiaoxinganling are highly distributed on the right side and the lower part of the clustering map in general. Similar to the results of the principal coordinate analysis, although the populations were not clearly divided, the clones of different populations had corresponding distribution ranges. For example, clones from Weihe had a small and relatively concentrated distribution range, which was consistent with the results of analysis of population diversity and the principal coordinate analysis, indicating that the genetic relationships among populations were similar and reflecting the degree of genetic differentiation within populations.

**Figure 6:**
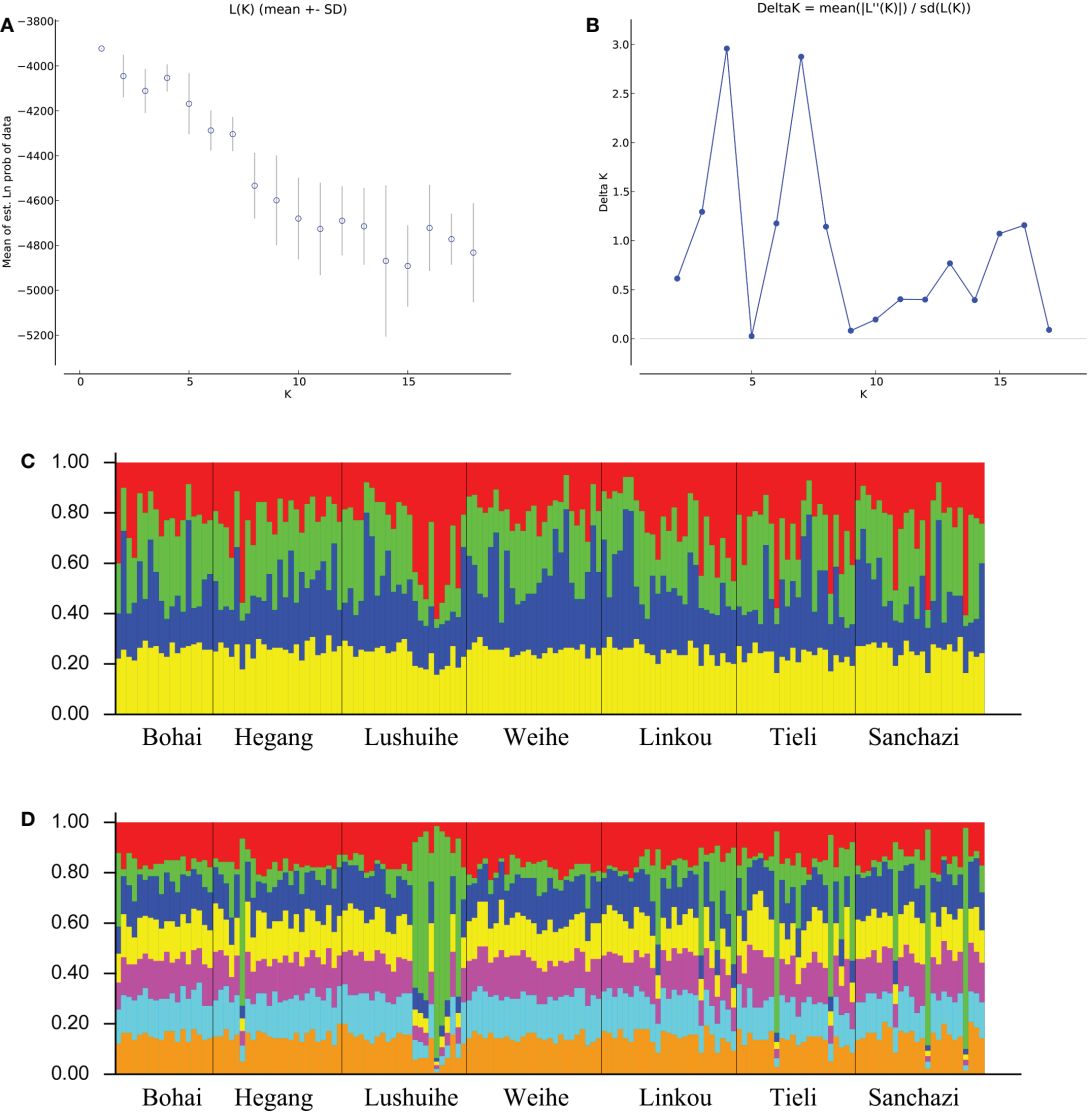
STRUCTURE analysis of Korean pine population. **(A)** Calculation of population structure using Mean LnP (K). **(B)** Relations between the optional number of cluster K and Delta K**(C)** Genetic structure map of 7 populations of Korean pine based on STRUCTURE analysis (K = 4). **(D)** Genetic structure map of 7 populations of Korean pine based on STRUCTURE analysis (K = 7).

Structure analysis was performed on all the reference materials. ΔK had a maximum value when K=4 and 7 in K=2-18 (**Figures 6A, B**), indicating that the 161 clones could be divided into 4 classes or 7 classes. The populations were not clearly differentiated and no individuals had 100% population affiliation in both cases (**Figures 6C, D**). However, the Lushuihe partial clones had a significantly high probability of occurrence in a certain population, indicating that the Lushuihe population partial clones had relative genetic independence, which was similar to the results of PCoA.

### 3.3 Fingerprint mapping construction

**Figure 7:**
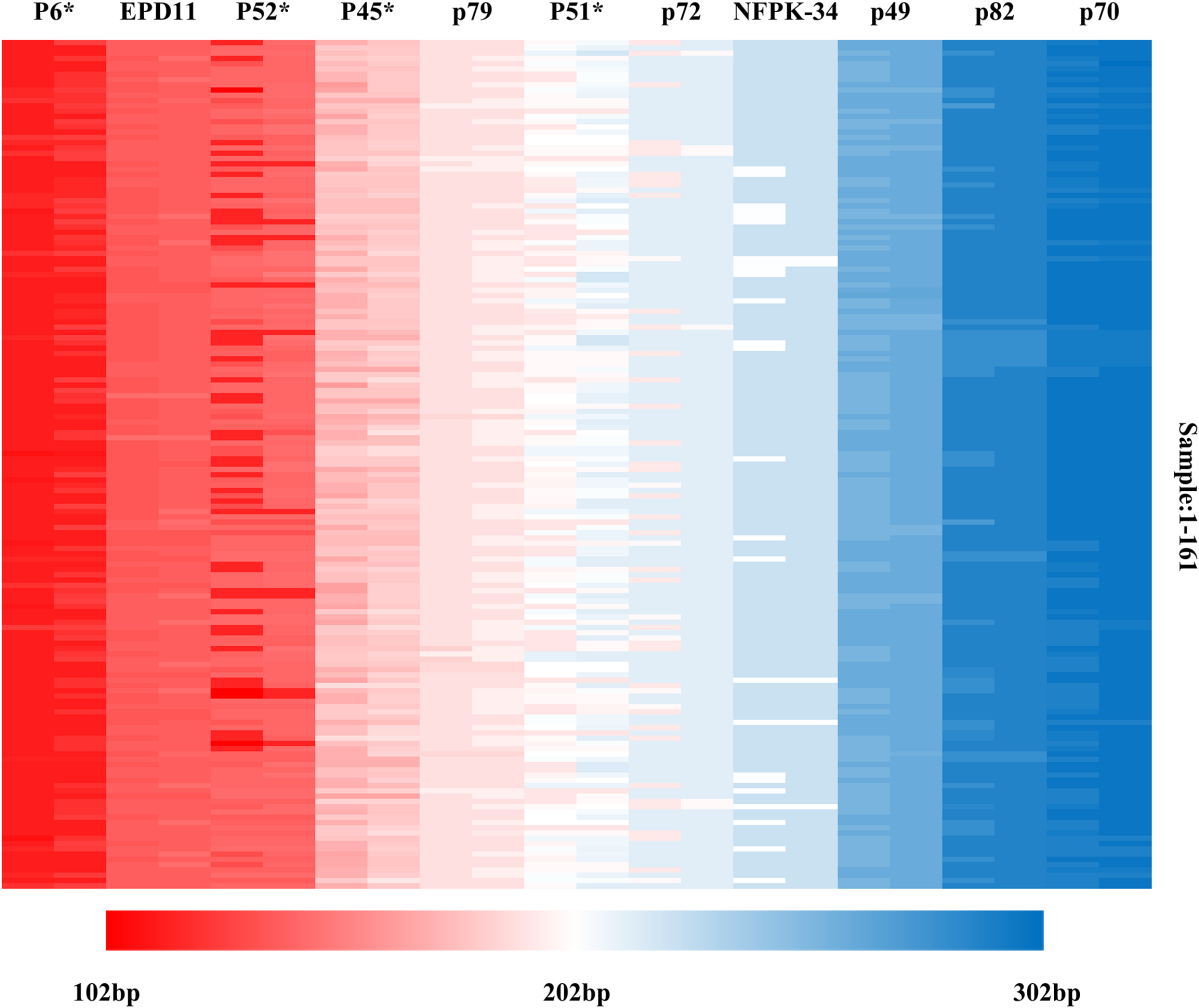
Molecular fingerprinting of Korean pine clones. (The different color blocks represent the corresponding allele fragment sizes).

Based on the genotyping data detected by 11 SSR markers, multiple locus matching analysis was performed in GenAlex 6.51 for 161 Korean pine clones. There is no identical genotype detected in two varieties, indicating that each of the 161 clones had its own unique SSR multi-locus genotype combination. The molecular markers were sorted according to the order of the target fragments from smallest to largest, and each marker consisted of two alleles. The molecular fingerprints of all 161 clones were generated according to the blocks with different color marking the different genotypes under each marker (**Figure 7**), with each color representing a variant locus information and each clone having a unique color block combination.

The genotyping data of each marker are indicated by letters respectively, and sorted in the order of amplified fragments from smallest to largest, and each clone gets its corresponding 22-digit letter code (**Supplementary Table S1**).

The name, location, source and genotyping data of each clone were uploaded to the QR code generation platform (https://cli.im/batch) to generate a unique QR code for each clone, which can be scanned to obtain specific information of the clone (**Supplementary Figure S1**).

## 4 Discussion

The genetic diversity of a population determines whether a population can adapt to a complex environment, and the higher the genetic diversity, the more adaptable the population is to different environments and the more resistant it is to shocks arising from environmental changes (Wachowiak et al., 2011). In order to develop a reasonable and effective breeding strategy, accelerate the process of genetic improvements of Korean pine, it is important to analyze the genetic diversity of Korean pine clone resources and evaluate the genetic structure of seed orchards by using SSR molecular marker. SSR markers have the advantages of codominance, stable amplification and good repeatability, which is a common method for genetic diversity analysis (Hao et al., 2017); at the same time, SSR molecular markers have strong specificity, clear bands and accurate data, which is suitable for the construction of fingerprint profile for a large number of resources (Park et al., 2009). Initially, we screened 11 Korean pine SSR loci, and the average values of *Na* and *He* for the 11 loci were 6.818, 0.514. *PIC* is an important parameter for expressing the degree of genetic diversity among plants, and its evaluation is beneficial to the establishment of plant gene pools and the acceleration of the breeding process (Avval, 2017). The average *PIC* of the SSR loci screened in this study was 0.475, showing moderate polymorphism (Botstein et al., 1980). Therefore, it is suitable for the genetic diversity evaluation of Korean pine breeding resources. The LD between 4 pairs of loci reached a significant level of P<0.001, but was not concentrated at one locus, and the results of PCoA showed that the molecular markers were highly independent, indicating that the screened loci were evenly distributed in the Korean pine genome and relatively independent in the process of transmission from generation to generation.

In order to elucidate the genetic variation among Korean pine populations, molecular variation analysis was conducted. The results showed that the genetic variation of Korean pine mainly originated from inter-individuals, accounting for 98%, and interpopulation variation accounted for only 2%. This indicates that the genetic differentiation within populations is much greater than between populations, which is consistent with the results of Feng et al. (Feng et al., 2006). The result is consistent with higher genetic diversity within populations and higher gene flow between populations. Therefore, we should pay attention to the selection of individuals within the population when the Korean pine population with high genetic diversity was constructed in the later stage, which is beneficial to the genetic improvement of Korean pine. The genetic diversity analysis of 7 populations revealed the differences in the level of genetic diversity among different populations, Tieli has the highest level of genetic diversity (*I*=1.087), the genetic diversity of Weihe population was low (I=0.915). Nevertheless, Weihe population is the only one with a fixed index (*F*) less than 0, indicating that the genetic diversity of this population is low, but there is no heterozygosity deficiency or inbreeding. Heterozygosity is often used to measure the degree of genetic variation and can provide useful information for the conservation of species (Schmidt et al., 2021). The results of this study based on SSR molecular markers showed that the overall *He* and *Ho* of 7 populations were 0.514 and 0.451. From a biological point of view, Korean pine is a monoecious, cross-pollinated plant that can generate new genotypes through genetic recombination, which is probably the main reason why Korean pine populations maintain a high genetic diversity. The *Ho* is smaller than *He* among these populations, except for the Weihe population, which indicating the presence of heterozygote deficiency, this may be due to inbreeding, non-random mating or disruption of population structure (Liao et al., 2019). Therefore, further analysis for the reason of heterozygote deficiency is necessary in future studies.

Genetic structure reveals the distribution patterns of genetic diversity between and within populations, reflects the adaptive potential of various species to their environment (Melo et al., 2014). Seven Korean pine populations in this study can be divided into 4 or 7 classes, and different populations are mismatched in classifications. Lushuihe population shows partial independence relatively, and the corresponding results were obtained by clustering results, which is consistent with the results of the principal coordinate analysis mentioned above. The results of interpopulation differentiation also show that the Lushuihe population has higher genetic differentiation and lower gene flow with other populations, which may be due to the relatively isolated population structure caused by the relatively unique geographical location of Lushuihe. Correlation analysis showed that there was no significant correlation between genetic distance and sources of superior tree’s geographical distance of Korean pine populations, which was also previously reported in Feng et al. (2009).

Screening and identifying the core SSR primer combinations suitable for variety identification is the key to constructing DNA fingerprinting. It is required that the core SSR primer combinations screened and identified have good marker polymorphism, and secondly, it is required that as few markers as possible are used to distinguish as many germplasms as possible. Construction of fingerprint profiles of Korean pine clones provides an important basis for the identification of resources from the 7 seed orchards. The DNA fingerprint profile of Korean pine clones based on SSR primer combinations can be directly used to identify the authenticity of clones in the 7 seed orchards, solving the long-standing problem of Korean pine clone identification. It is important for the selection and breeding of Korean pine clone in these 7 seed orchards. The critical point to ponder, the established fingerprint panel or Korean pine clonal identification was based on 7 seed orchards in northeast China. It does not cover the distribution range of the species which also can be found in Korea, Russia, Mongolia & Japan. Hence, this clonal identification tool developed will solely useful within China (limited to the resources from the 7 seed orchards).

In this study, 11 SSR markers were screened out, which could be used for the construction of fingerprints of Korean pine clones and the evaluation of genetic structure of the population. Genetic analysis of 7 populations of Korean pine using 11 SSR primers revealed the level of genetic diversity and genetic differentiation among and within populations. According to the genetic characteristics of Korean pine clone populations, the development of corresponding breeding strategies can maximize the breeding potential of Korean pine seed orchards and provide a scientific basis for the subsequent development and utilization of Korean pine germplasm. The DNA fingerprints of 161 Korean pine clones were constructed, which is an effective strategy for the identification of Korean pine clone, it will provide strong DNA evidence for identification of variety and superior seed validation.

## Data availability statement

The original contributions presented in the study are included in the article/**Supplementary Material**. Further inquiries can be directed to the corresponding author.

## Author contributions

Conceptualization: PY and HZ. Methodology: XQ. Validation: KF. Resources: ZX. Writing-original draft preparation: PY. Writing-review and editing: LZ. All authors contributed to the article and approved the submitted version.
