# Supplementary figures and images for: Genetic diversity analysis and fingerprint construction of Korean pine (Pinus koraiensis) clonal seed orchard
Source: Front Plant Sci. 2023 Jan 16;13:1079571. doi: 10.3389/fpls.2022.1079571 (PMC9886227; doi:10.3389/fpls.2022.1079571)

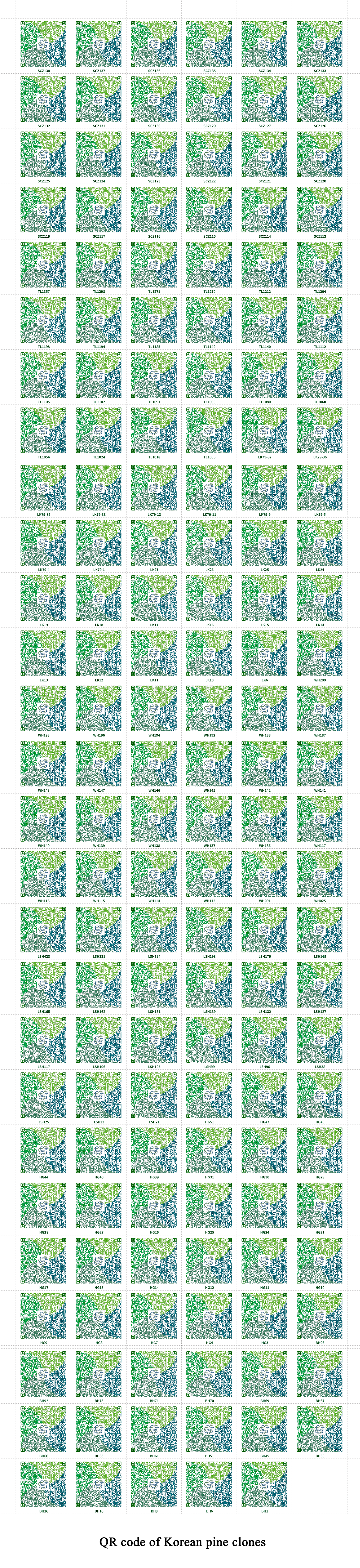

Supplement: Supplementary file 1 [file Image_1.tif]
