# Supplementary material for: Genetic diversity analysis and fingerprint construction of Korean pine (Pinus koraiensis) clonal seed orchard
Source: Front Plant Sci. 2023 Jan 16;13:1079571. doi: 10.3389/fpls.2022.1079571 (PMC9886227; doi:10.3389/fpls.2022.1079571)
Supplement: Supplementary file 2 [file Table_1.docx]

**Table S1 Letter code of Korean pine clones**

| Clones | Code | Clones | Code | Clones | Code | Clones | Code | Clones | Code | Clones | Code | Clones | Code |
| --- | --- | --- | --- | --- | --- | --- | --- | --- | --- | --- | --- | --- | --- |
| BH1 | BBBCACCCAACCAAAHHLGJCJ | HG3 | BDBBAACCAACDAAACGIIIEF | LSH21 | BBBBAAACAACDABFFEGANAD | WH025 | BDBBAAACAACDAAHHGPFJDD | LK6 | BDBBACACAACDAAHHIIHIDD | TL1006 | BDBBACAEAACCAAFHHHJNBJ | SC113 | BBBBAACCAACDAAHHLLBNBF |
| BH6 | BBBBAACCAACCAAHHGGEHDD | HG4 | BBBBACCCAACCAAFFFHIIEJ | LSH22 | BBBBAAACAACDAAHHHLAGDF | WH091 | BDBBAACCAACDAAAHHHFJDD | LK10 | BDBBAACCADACAAHHHHNNHH | TL1018 | BDBCAAAAAACDAACHEGCDCE | SC114 | BDBBAACCAACCAAHHGIBNDD |
| BH8 | BDBBBCCCACCCAAHHGIAECC | HG7 | DDBCBCACAACCAACHFHJJCD | LSH25 | BDBBAACCAADDAAHHJJFNJJ | WH112 | BDBBAACCAAAAAADHHHDIDJ | LK11 | DDBBAACCAABCAAAHGMHNDD | TL1024 | BDBDAAAEAACCAABHFGCDCD | SC115 | BDBDAACCAACCABHHHIFKBD |
| BH16 | BBBCAAACAAADAACHFGJJDJ | HG8 | BDBBBCCCAACCAAADHHJJEJ | LSH38 | ABBBAAACAADDAAHHGIDKDD | WH114 | BDBBAACCAACDAACHMMDIDJ | LK12 | DDBBACCCAADDAAHHIIEHHH | TL1054 | BBBDAADDAAACAAHHGHIIDD | SC116 | BBBBAACCAACDABHHFLIJDD |
| BH26 | BDADAACCAACCAAHHGJFHDH | HG9 | BBBBAAAAAACCAAAHDFNNDD | LSH96 | AABBAACCAACCAAFHFLIKCD | WH115 | BDBBACCCAACDAAHHIIJJDD | LK13 | BDBBAAACAADDABHHJJENDD | TL1068 | BBBDAADDABCCAAHHILIIDG | SC117 | BDBBACCCACACAADHJLEEDD |
| BH38 | BDBCAAACAACCAAHHHHGJDD | HG10 | BBBCAAACAACDAAHHELJJJJ | LSH99 | BDBBAAACAACCABFHGLEFDD | WH116 | BDBBAAACAACDAAHHFICKDF | LK14 | BBBDABACAADDAAHIJJKKDD | TL1080 | BBBBAACCABDDAAHHEJEIDD | SC119 | BDBDAAAAAACCABFHFGNNCC |
| BH45 | BDBBAACCAACDAADHGKHNDD | HG11 | BBBCAAAAACCCABHHHMIIDF | LSH105 | BDBBAACCAACDAAHHGLCJBD | WH117 | BBBBAADDAACDAAFHKMFFCH | LK15 | BBBDAACCAACCAACHFHFNDD | TL1090 | BBBBAACCACCCBBDHGNNNDJ | SC120 | BDBDAAACAADDAAHHNNJNCC |
| BH51 | BDBCAACCAAACAADHGKHNDD | HG12 | BBBBACCCAACDABHHGGEJCJ | LSH106 | BBBBACCCAACDAAFHGIJMDE | WH136 | BDBBAAACAACCAAHHILCJDD | LK16 | BBBBAACCCCCDAAHHHHFNCC | TL1091 | BBBDACCCACCDAADHCCCJEJ | SC121 | BDBDBCCCAACCAADHEEJNEE |
| BH61 | BBBBACACAACDAADHGPGJCD | HG14 | BDBCACCCAACCAAFHHHFMDD | LSH117 | DDBBAACCAACDAAFHGLMNCD | WH137 | BDBBAAACAACDAAHHEJDIEE | LK17 | BBBBAAACCCDDABFFGMCGCC | TL1102 | BDBDABACACACAAHHGJHKJK | SC122 | BDBCBCACABCCBBDHHKIJCF |
| BH63 | DDCDAACCACCDAABFGNFHCK | HG15 | BDBCACCCAACCAAFHHPFMDD | LSH127 | DDBBAACCCCCDAAHHHHJNDI | WH138 | BBBBAAACAACDAACHBLINHJ | LK18 | BDBCAACCAACCAAHHIICGDJ | TL1105 | BDBDAAACAACDAADHFLJJJK | SC123 | BDBBAACCAACCAADHHLGICC |
| BH66 | BBBCAACCACCCAADFHHEJCD | HG17 | BBCDAACCAACDAAFHGIJNDD | LSH132 | DDBDBCACAACDAAAHGHJKCD | WH139 | BBBBAAACAAAAAACHILHJCJ | LK19 | BDBCACCCAACDAAGHEGJKDJ | TL1112 | BDBDAAACAACDAAHHFLJJDD | SC124 | BBBBAACCAACDAABHGGEIFF |
| BH67 | BBBBAAACAABDAADDLLJJDI | HG21 | BBBBACCCAACDAAFFFHDJEJ | LSH139 | BBCCAAACCCCDAAHHJLFNJJ | WH140 | BBBBAACCAACDAAHHCFFFCH | LK24 | BBBBAAACAACDAACHGIHJCD | TL1140 | BBBDABACAACCAAHHGGKNEE | SC125 | BBBDAAACACACABHHGLIIEE |
| BH69 | BBCDAAAAADCCAABHHHJJDF | HG24 | BDCDAACCAACDAAAFIIBFBD | LSH161 | BDCCAACCCCCDAAAFLLAJBJ | WH141 | BDBBAACCACCDAAIICFFFCH | LK25 | BBBDACACAACCAAHHGIHJCD | TL1149 | BDBCACACAADDAADHDGKNDD | SC126 | BDBDAACCACCCAAHHEGNNDD |
| BH70 | BDBBAACCAACDAADHGJNNBD | HG25 | BBBCAAACAACDABCFIIINDE | LSH162 | BDCCAAACCCCDABDHGGAJCJ | WH142 | BDBBAAACACCDABHHGGHJDJ | LK26 | BBBDAACCAADDAAADFOEICD | TL1185 | BBBBAACCAADDAAHHGGFHDD | SC127 | BBBCACBCACCCAAHHCGJJEE |
| BH71 | BBBCAACCAACCAAHHFNGICD | HG26 | BDBBAACCAACDABAIIICFEJ | LSH165 | BDCCAACCCCCDABHHDHEFDF | WH145 | BDBBACCCACDDAAHHGGEIBJ | LK27 | BBBBAAACAACDAADHFOJNJJ | TL1194 | BBDDAACCACCDBBHHILFHDG | SC129 | BDDDACCCAACCAADJHHFFDD |
| BH73 | DDBBAAACAACCAABHHHDJEH | HG27 | DDBBAACCACACABFHFFHMDJ | LSH169 | BBCCACACCCCDAADHGLJJEE | WH146 | BDBBACCCAACDAAFHKMJKBD | LK79-1 | DDBBAACCAAACAABHFLCGJJ | TL1198 | BBBBACACACCCAAHHDIEIDD | SC130 | BDBDAACCAACDAAAHGLFKDD |
| BH92 | BDCDAACCAACDAADHGLINBD | HG28 | BBBBAACCAACDABAHGGHJJJ | LSH179 | BDCCAAACCCCDAAHHFKJJEJ | WH147 | BBBBAACCAACDAAAHGJCJDJ | LK79-4 | DDBBAACCAACCAAAHFLEIDD | TL1204 | BBBCAACCABCCAAHHDINNDJ | SC131 | BDDDAACCAACCABBFFLEHCC |
| BH93 | BBBDAACCAACCAAHHGLFJBD | HG29 | DDBBAACCACCCAADHHIINDH | LSH193 | BBCCAACCCCCCAAHHGIJJCH | WH148 | BDBBACACAADDAAHHHICJDD | LK79-5 | BDBBAAACAACCAADIGNJNDD | TL1212 | BDBCACACABCDAADHBCCJEJ | SC132 | BDDDAAACAAACAAHHGNFJDI |
|  |  | HG30 | BBBBAAACAACCAAHHFHNNDD | LSH194 | BBBDAACCACCCAAHHMMGJCD | WH187 | BDBBAACCAACDAAHIJJFKDF | LK79-9 | BBBCAACCAACCAAHHCGCGDJ | TL1270 | BDBDAAACAACCAADHGIHKJK | SC133 | BDBBAACCACCDAAHHHMEKEE |
|  |  | HG31 | BDBBAACCAACDAACHGLHNJJ | LSH331 | BDBDAAACACCCAAHHGGJJBE | WH188 | BDBBABACAACDAAFHHLFKDE | LK79-11 | BBBBABCCAACCAAHHFLEICC | TL1271 | BDBCAACCAACCAADHFLJJCJ | SC134 | BDBDAACCAAACAAHHHMJJDI |
|  |  | HG39 | BBCDAAACAAADAADHGJEHDJ | LSH428 | BDBBACACAACDAAAHCGKNDJ | WH192 | BDBBAACCAACDAACHHLHJCJ | LK79-13 | BBCDAAACACACAADHGNJNCC | TL1298 | CDBBAADDCCBDAAHHELIIEE | SC135 | BBCCACACAACCAAHHGLJJEE |
|  |  | HG40 | BDBBAAACAACCAAFHFHENDI |  |  | WH194 | BDBBACCCAACDAAHHHNHJCH | LK79-33 | BBCDACACAACCAAABCGCGDJ | TL1357 | BDBDAACCCCCCAAGGLLFHEE | SC136 | BBDDAAACAACCAABFFLEHCC |
|  |  | HG44 | BBBBAACCAACDAAADDLKKBE |  |  | WH196 | BDBBAACCAACDAAHHEGCJDJ | LK79-35 | BBBDAAACAACDAADHFGJNDJ |  |  | SC137 | BDBDABCCACCCABHHAGHHFF |
|  |  | HG46 | BBCDABACAACCBBHHHHJNBC |  |  | WH198 | BDBBAAACAACDAAAHFJCKDI | LK79-36 | BBBDABACAACDAABHGICNEE |  |  | SC138 | BDBDAACCAACCAAADGLCJDD |
|  |  | HG47 | BBBBAAACAACCBBHHHHJNBC |  |  | WH200 | BDBBAAACAAADAAFHGGFFJJ | LK79-37 | BBBDACCCAACCAAHHGIJNEE |  |  |  |  |
|  |  | HG51 | BDBBAAACAACDABBHGMHIDD |  |  |  |  |  |  |  |  |  |  |
